# Disturbed glycolipid metabolism activates CXCL13-CXCR5 axis in senescent TSCs to promote heterotopic ossification

**DOI:** 10.1007/s00018-024-05302-3

**Published:** 2024-06-17

**Authors:** Yuyu Chen, Jinna Wu, Chipiu Wong, Wenjie Gao, Xiangdong Qi, Hang Zhou

**Affiliations:** 1 Department of Plastic Surgery, Zhujiang Hospital, Southern Medical University, Guangzhou, 510515 China; 2 Department of Breast Surgery, Affiliated Cancer Hospital & Institute of Guangzhou Medical University, Guangzhou, 510095 China; 3 Department of Orthopaedics, Sun Yat-sen Memorial Hospital of Sun Yat-sen University, Guangzhou, 510120 China

**Keywords:** Heterotopic ossification, Glycolipid metabolism, Cellular senescence, CXCL13

## Abstract

Heterotopic ossification (HO) occurs as a common complication after injury, while its risk factor and mechanism remain unclear, which restricts the development of pharmacological treatment. Clinical research suggests that diabetes mellitus (DM) patients are prone to developing HO in the tendon, but solid evidence and mechanical research are still needed. Here, we combined the clinical samples and the DM mice model to identify that disordered glycolipid metabolism aggravates the senescence of tendon-derived stem cells (TSCs) and promotes osteogenic differentiation. Then, combining the RNA-seq results of the aging tendon, we detected the abnormally activated autocrine CXCL13-CXCR5 axis in TSCs cultured in a high fat, high glucose (HFHG) environment and also in the aged tendon. Genetic inhibition of CXCL13 successfully alleviated HO formation in DM mice, providing a potential therapeutic target for suppressing HO formation in DM patients after trauma or surgery.

**Supplementary Information:**

The online version contains supplementary material available at 10.1007/s00018-024-05302-3.

## Introduction

Heterotopic ossification (HO) refers to the pathological formation of ectopic bone in the tendon, ligament, muscle, and other soft tissues [1]. According to the different etiologies, HO can be divided into acquired non-genetic forms and inherited genetic forms. As the most common type of HO, acquired HO commonly occurs after musculoskeletal trauma or neurogenic trauma. Genetic HO, which results from gene mutation, consists of fibrodysplasia ossificans progressiva (FOP) [2, 3], progressive osseous heteroplasia (POH) [4], and Albright inherited osteodystrophy (AHO) [5]. Patients with HO often develop chronic pain, unhealed wounds, and restricted motion, leading to a diminished quality of life [6]. However, due to the uncertainty of the pathology, there is still no effective therapy for HO.

Multiple risk factors have been identified for the initiation and progression of HO, including obesity, mechanical stress, and infection. In recent research, various epidemiological studies have suggested a possible connection between diabetes mellitus (DM) and HO. For example, obesity-related genes were detected in the pathogenesis of ossification of ligamentum flavum (OLF) [7]. Similarly, taking advantage of genome-wide association studies (GWASs) data of ossification of the posterior longitudinal ligament (OPLL) also indicated a genetic correlation between OPLL and body mass index (BMI) and DM [8]. In addition, increased thickness and calcified fibers were detected in the diabetic tendon, resulting in decreased tensile tolerance and a higher risk of tendon rupture [9, 10]. This also increased the stiffness of the tendon and led to a limited range of joint motion [11, 12]. However, despite the connection between DM and HO, there is still no in vivo study exploring this mechanism.

Lineage-tracing studies have examined which specific mesenchymal progenitor subsets are the main contributors to HO. The *Scx*^*+*^ tendon-derived stem cells (TSCs) have been proven to be progenitor cells of tendon and ligament HO. Multiple pathological factors, including hypoxia, inflammation, and angiogenesis, have been identified as contributing to the abnormal osteogenic differentiation of TSCs. After trauma, inflammation caused by macrophage infiltration changes the commitment of progenitor [13], and the local hypoxic environment also induces hypoxia-induced factor 1 alpha (HIF1α) to mediate the endochondral ossification of progenitor [14], which finally increases the differentiation of progenitors. During the regeneration phase, angiogenesis promotes the osteogenic differentiation of progenitors [15]. Previous studies revealed that inflammation and abnormal angiogenesis were both important pathologic changes in DM patients, but how the aberrant metabolic environment changes the differentiation of TSCs is still unknown.

In the current study, a higher occurrence rate of tendon and ligament HO was observed in DM patients. The DM mice model was then established and exerted with Achilles tenotomy. Similar to the DM patients, larger and more extensive tendon HO was detected in the DM mice. Mechanically, disordered glucolipid metabolism promoted the senescence and osteogenesis of TSCs by regulating the level of chemokine C-X-C motif ligand 13 (CXCL13). Furthermore, local injection of adeno-associated virus (AAV) was exerted to knock out *Cxcl13*, and tendon HO in DM mice was significantly rescued. Our study provides new insight into the underlying mechanism of tendon HO in DM and suggests a potential target for the treatment of HO in DM patients.

## Results

### DM patients and DM mice were more susceptible to developing tendon and ligament HO

**Fig. 1:**
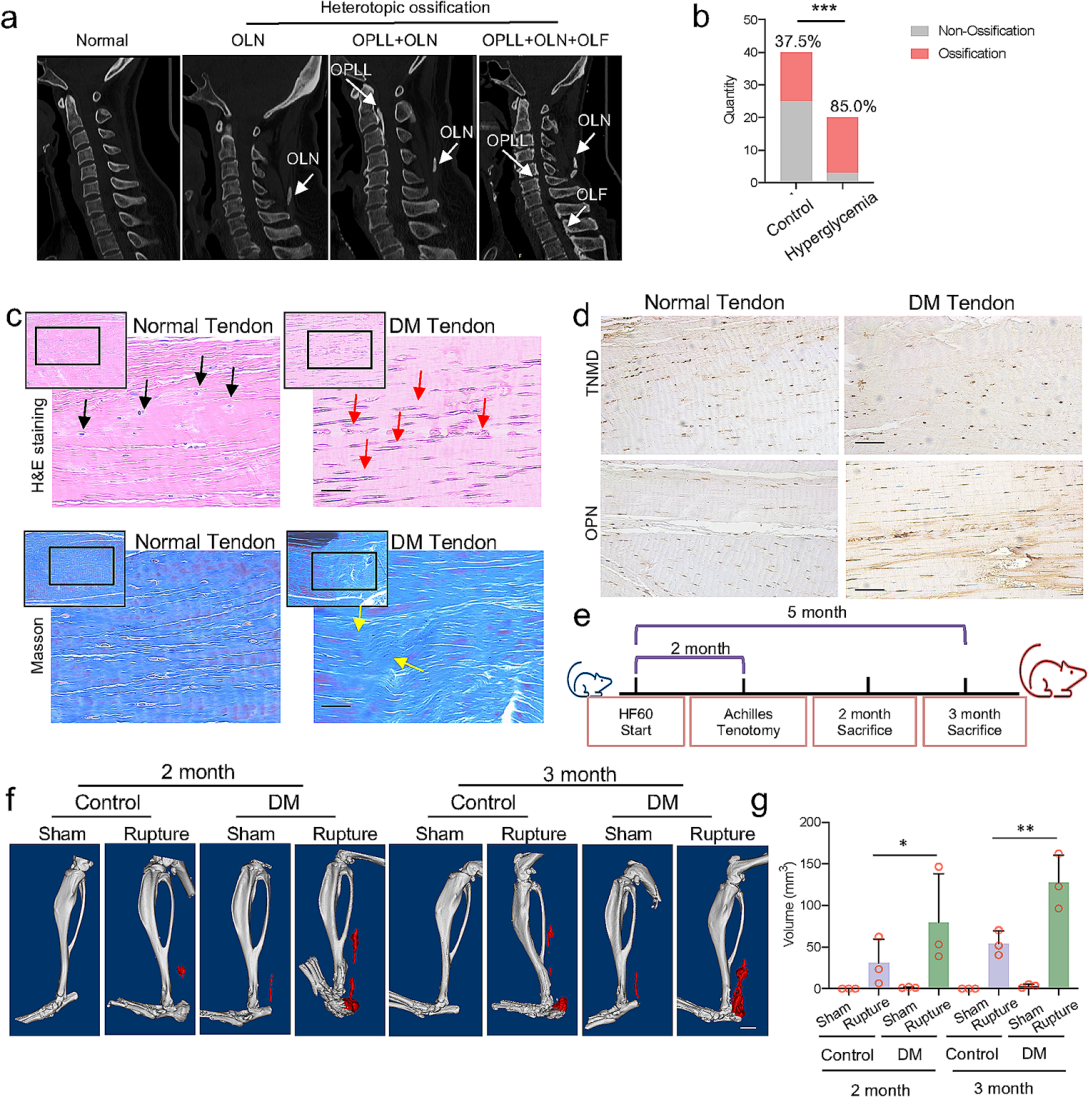
DM patients and DM mice were more susceptible to developing tendon and ligament HO. **a**. Sagittal view of CT scanning of patients suffering from HO in different ligaments; OLN: Ossification of ligamentum nuchae; OPLL: Ossification of posterior longitudinal ligament; OLF: Ossification of ligamentum flavum. **b**. Occurrence rate of heterotopic ossification in control patients and hyperglycemia patients. **c**. H&E staining (upper lane) and Masson trichrome staining (bottom lane) of the normal tendon and DM tendon. Black arrows indicate normal tendon cells. Red arrows indicate apoptotic tendon cells. Yellow arrows indicate the anomaly-aligned fibers in the DM tendon. Scale bar = 100 μm. **d**. TNMD staining (upper lane) and OPN staining (bottom lane) of normal tendons and DM tendons. Scale bar = 1 mm. **e**. Diagram of the construction of DM mice and tenotomy. **f**. CT scanning of control mice and DM mice receiving sham or tenotomy for 2 or 3 months. The red part indicates ectopic bone. Scale bar = 2 mm. **g**. The volume of ectopic bone detected in control mice and DM mice which received sham or tenotomy for two or three months. The *p*-value was calculated by chi-square test (b) or one-way ANOVA, followed by Tukey’s multiple comparisons tests (g). Data are shown as mean ± SD. *p < 0.05; **p < 0.01; ***p < 0.001

First, we collected patients suffering from cervical spondylosis, including cervical radiculopathy and cervical myelopathy, and analyzed their CT scanning images (Fig. 1a). After controlling the variables, including age, sex, and body mass index (BMI) (Supplementary Table 1), we divided the patients into two groups according to the hemoglobin A1C (HbA1C): Control group (HbA1C < 6%) and Hyperglycemia group (HbA1C ≥ 6%). Then, we detected 37.5% of patients with HO in the Control group and 85% in the Hyperglycemia group (Fig. 1b), which suggests that hyperglycemia may be a risk factor for HO. We also collected tendon tissue from patients diagnosed with DM and from patients with normal blood glucose levels. A scattered cavity was observed in the DM tendon (Fig. 1c, upper lane), suggesting that increased cell apoptosis occurred in the tendon tissue of the DM patients. Unlike the normal tendon, the fiber of the DM tendon exhibited significant curly and disorder (Fig. 1c, bottom lane). Then, we exerted the staining of the markers of tenogenesis, tenomodulin (TNMD) [16], and bone formation, osteopontin (OPN) [17], and the results showed that, even though no significant difference of TNMD between the normal tendon and DM tendon, the OPN positive cells increased in DM tendon (Fig. 1d), suggesting that the anomaly osteogenic change of tendon occurred in DM tendon.

In order to investigate the mechanism underlying tendon HO in DM, we constructed the type II DM by feeding the mice with 60% high fat fodder (HF60) for two months [18]. Then, we exerted Achilles tenotomy to induce the occurrence of HO (Fig. 1e) [1]. Long-term induction resulted in a significant increase in body weight and blood glucose in the DM group (Supplementary Fig. S1a-b). Comparing to the Control mice, the DM mice exhibited reduced glucose tolerance, decreased sensitivity to insulin, elevated serum insulin level, and the damaged reaction to elevated blood glucose (Supplementary Fig. S1c-d) [19]. In contrast to the obese appearance, a slightly reduced thickness of the ligamentum patellae and ligamentum caudale was observed in DM mice (Supplementary Fig. S1e). Two and three months after the Achilles tenotomy, a micro-CT was taken of the mice to measure the volume and bone mineral density of the tendon HO. For mice that received tendon ruptures, an increasing level of HO volume was detected in DM compared to the control group (Fig. 1f–g), while the bone mineral density of the ectopic bone showed no difference (Supplementary Fig. S2a–b). Interestingly, spontaneous tendon HO was also detected in DM mice who received sham surgery (Fig. 1f–g). Taken together, the above results indicated that DM mice were more susceptible to forming tendon HO, even without trauma.

### Tendon in DM mice exhibited a stronger osteochondrogenic change

**Fig. 2:**
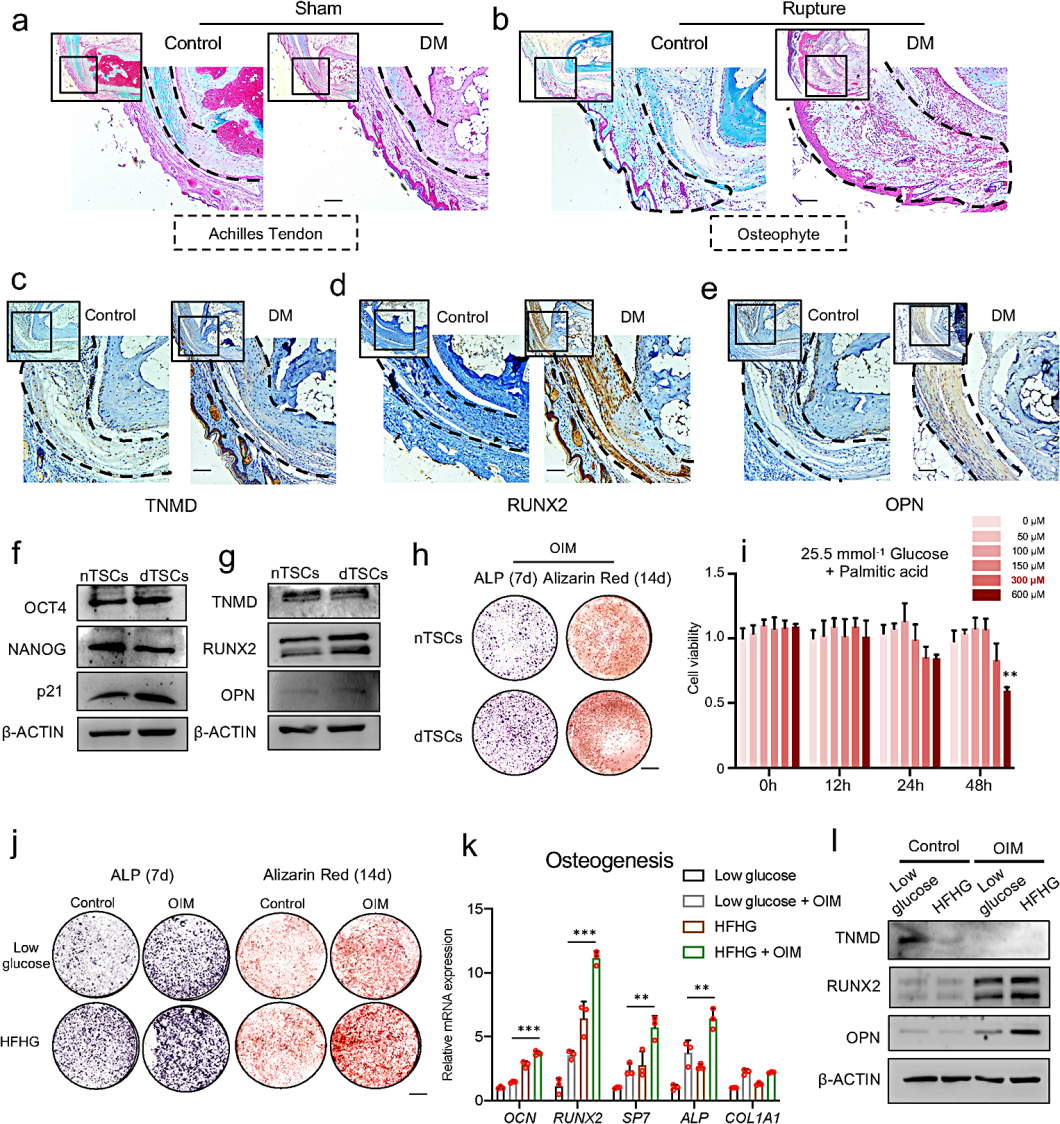
Tendons in DM mice exhibited a stronger osteochondrogenic change. **a**–**b**. Safranine O staining of the tendons of Control mice and DM mice receiving sham (a) or tenotomy (b). Dashed boxes indicate the Achilles tendon (a) or osteophyte (b). Scale bar = 100 μm. **c**–**e**. TNMD (c), RUNX2 (d), and OPN (e) staining of the tendons of the Control mice and DM mice. Scale bar = 100 μm. **f**. Protein levels of OCT4, NANOG, and p21 in normal TSCs (nTSCs) and diabetic TSCs (dTSCs). **g**. Protein levels of TNMD, RUNX2, and OPN in nTSCs and dTSCs. **h**. ALP staining and alizarin red staining of nTSCs and dTSCs cultured in OIM. Scale bar = 1 mm. **i**. Relative cell counting of TSCs cultured in a 25.5 mmol^− 1^ glucose medium, adding different concentrations of palmitic acid. **j**. ALP staining and alizarin red staining of TSCs cultured in low glucose medium and HFHG medium under control or OIM conditions. Scale bar = 1 mm. **k**. The expression of mRNA relevant to osteogenesis of TSCs cultured in low glucose medium and HFHG medium under control or OIM conditions. The expression of *Actb* was used as the internal control. **l**. Protein levels of TNMD, RUNX2, and OPN of TSCs cultured in low glucose medium and HFHG medium under control or OIM conditions. The *p*-value was calculated by one-way ANOVA, followed by Tukey’s multiple comparisons tests (i, k). Data are shown as mean ± SD. **p < 0.01; ***p < 0.001

To further study the pathological changes in the DM tendon, we compared the Achilles tendons of the Control and DM mice. By using safranine O staining, enhanced chondrogenic changes and decreased collagen levels were detected in both DM mice with and without trauma (Fig. 2a–b). We then compared the tenogenesis or osteogenesis by IHC. The signal of TNMD decreased in the Achilles tendon of DM mice (Fig. 2c), while the marker of osteogenesis, such as RUNX2 and OPN, increased significantly (Fig. 2d-e), suggesting that metabolic disturbance promoted the osteogenesis of tendon cells. Furthermore, TSCs were isolated from both DM (dTSCs) and Control mice (nTSCs) patellar ligaments, and the expression of stemness (OCT4 and NANOG), tenogenetic, and osteogenetic markers were detected. Slightly decreased expression of NANOG was detected in the dTSCs (Fig. 2f). Similar to the IHC results, decreased expression of TNMD and increased expression of RUNX2 and OPN were observed in the dTSCs (Fig. 2g). Then, two different types of TSCs were treated with osteogenic-induced medium (OIM) to induce osteogenic differentiation. Increased alkaline phosphatase (ALP) activity at Day 7 and increased alizarin red staining intensity at Day 14 were detected in the dTSCs group (Fig. 2h), indicating enhanced osteogenesis in the dTSCs.

To imitate hyperglycemia and obesity in DM mice, we cultured the TSCs with low glucose (5.5 mM glucose + DMSO) or high fat, high glucose (HFHG, 25.5 mM glucose + palmitic acid), respectively. First, we performed a CCK-8 assay to confirm the safe concentration of palmitic acid, and 300 μM palmitic acid showed no significant cytotoxicity for TSCs (Fig. 2i). Thus 300 μM palmitic acid was exerted in the following experiment. By using ALP and alizarin red staining, an increased level of osteogenesis was detected in TSCs cultured with HFHG medium. The trend was more significant under OIM conditions (Fig. 2j).

The qPCR using mRNA extracted from TSCs cultured with different mediums confirmed decreased tenogenesis, manifesting decreased expression of *Scx* and *Tnmd* in the HFHG medium, while the expression of *Mkx* showed no significant change (Supplementary Fig. S3). Consistent with the above results, increased osteogenesis was detected in TSCs cultured in HFHG medium, especially under OIM conditions (Fig. 2k). The above change was also confirmed at the protein level (Fig. 2l). Thus, we discovered that disordered glycolipid hemostasis damaged tenogenesis and enhanced the osteogenesis of TSCs.

### Disturbed glycolipid metabolism promoted TSC senescence and enhanced osteogenesis

**Fig. 3:**
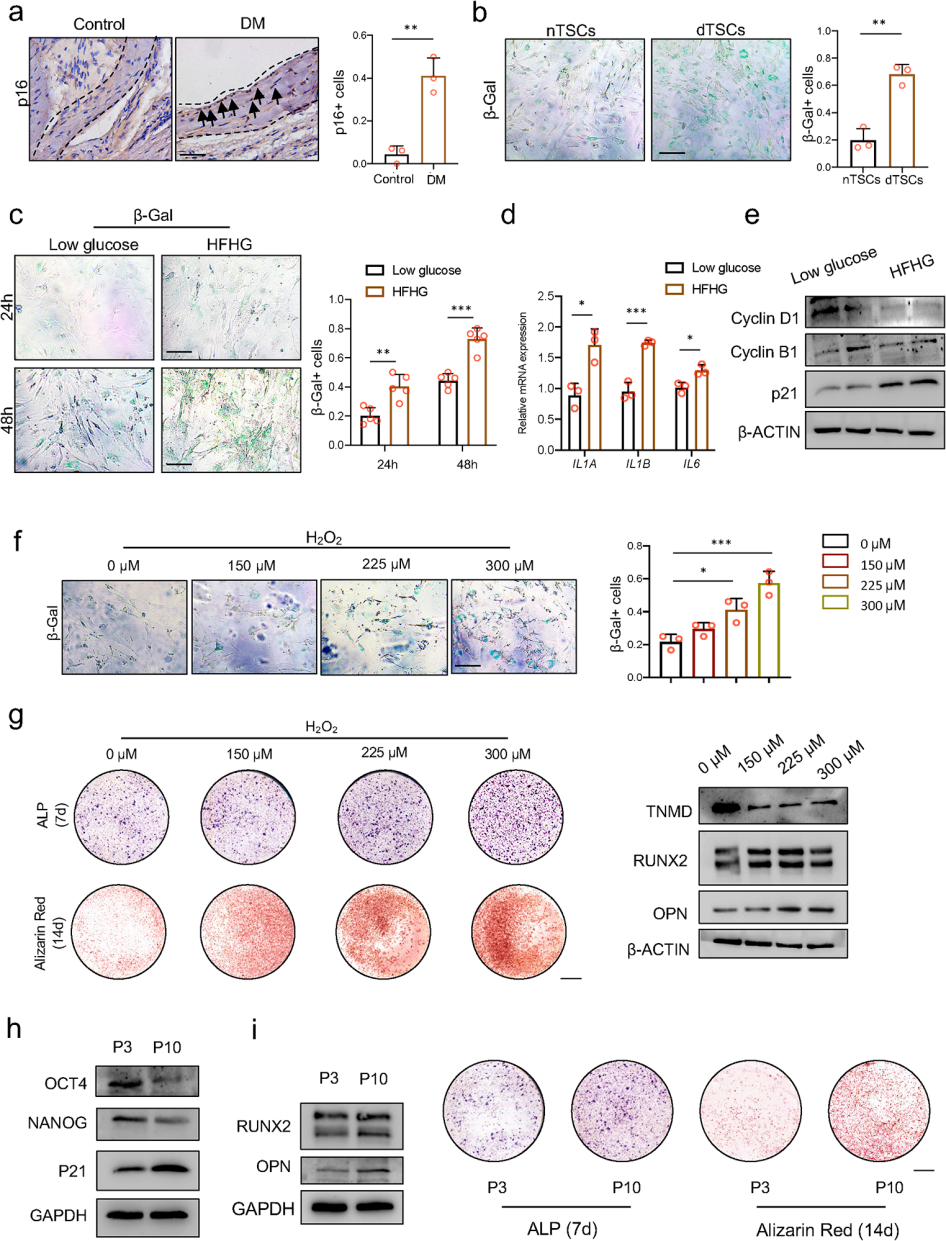
Disturbed glycolipid metabolism promoted TSC senescence and enhanced osteogenesis. **a**. The p16 staining of the Achilles tendons of Control mice and DM mice and their quantification. Scale bar = 100 μm. **b**. The β-Gal staining of nTSCs and dTSCs and their quantification. Scale bar = 50 μm. **c**. The β-Gal staining of TSCs cultured in low glucose medium and HFHG medium and their quantification. Scale bar = 50 μm. **d**. The expression of mRNA relevant to SASP of TSCs cultured in low glucose medium and HFHG medium. **e**. Protein levels of cyclin D1, cyclin B1, and p21 of TSCs cultured in low glucose medium and HFHG medium. **f**. The β-Gal staining of TSCs cultured in 0, 150, 225, or 300 µM H_2_O_2_ and their quantification. Scale bar = 50 μm. **g**. ALP staining and alizarin red staining (left) and the protein levels of TNMD, RUNX2, and OPN (right) of TSCs cultured in 0, 150, 225, or 300 µM H_2_O_2_. Scale bar = 1 mm. **h**. Protein levels of OCT4, NANOG, and p21 of TSCs after 3 times passage (P3) and 10 times passage (P10). **i**. Protein levels of RUNX2 and OPN (left) and ALP staining and alizarin red staining (right) of P3 TSCs and P10 TSCs. Scale bar = 1 mm. *The p*-value was calculated by a two-tailed, unpaired Student’s t-test (a–d) or one-way ANOVA followed by Tukey’s multiple comparisons tests (f). Data are shown as mean ± SD. *p < 0.05; **p < 0.01; ***p < 0.001

As disturbed glycolipid metabolism has been shown to aggravate cell senescence in multiple tissues, we speculated that tendon HO occurring in DM mice may result from the senescence of TSCs. Compared with the Control mice, the senescent marker p16-positive cells significantly increased in the DM mice tendons (Fig. 3a) and in the tendons of the DM patients (Supplementary Fig. S4). We performed senescence-associated, beta-galactosidase (SA-β-Gal) staining to label the senescent cells. As expected, more β-Gal positive cells were detected in dTSCs (Fig. 3b), along with the increased expression of another senescent marker, p21 (Fig. 2f). We also exerted SA-β-Gal staining in TSCs cultured with low glucose medium or HFHG medium. Compared with the low glucose medium, the TSCs cultured in the HFHG medium exhibited significant cell senescence, and cell senescence increased alone with cultural time (Fig. 3c). Except for the enhanced β-Gal activity, the senescent cells also exhibited a senescence-associated secretory phenotype (SASP), manifesting the upregulating expression of pro-inflammation factors, including IL-1 and IL-6. We compared the SASP of TSCs cultured under low glucose medium and HFHG medium for 48 h and detected increased mRNA levels of *IL1A*, *IL1B*, and *IL6* (Fig. 3d). Due to the cell senescence that occurred in TSCs cultured with HFHG medium, a significant cell cycle arrest was detected, manifesting decreased expression of cyclin D1 and cyclin B1 and increased expression of p21 (Fig. 3e). To examine whether senescent TSCs were prone to osteogenesis, two different senescence models were used. First, we treated TSCs with different concentrations of H_2_O_2_ to induce cell senescence. The H_2_O_2_ concentration of 225 µM or 300 µM was sufficient to induce TSC senescence (Fig. 3f). After being cultured with OIM, the senescent TSCs exhibited damaged tenogenesis and enhanced osteogenesis (Fig. 3g). Stem cells lose their regenerative ability and experience spontaneous senescence after serial passages. Thus, we detected decreased stemness and increased senescence of TSCs in Passage 10 (P10) (Fig. 3h), and, similar to the induced senescent cell model, the osteogenesis of P10 was stronger than that of P3 (Fig. 3i). To sum up, our data indicated that disordered glycolipid metabolism aggravated TSC senescence and enhanced osteogenesis.

## Increased levels of CXCL13 in DM mice promote the osteogenesis of TSCs

**Fig. 4:**
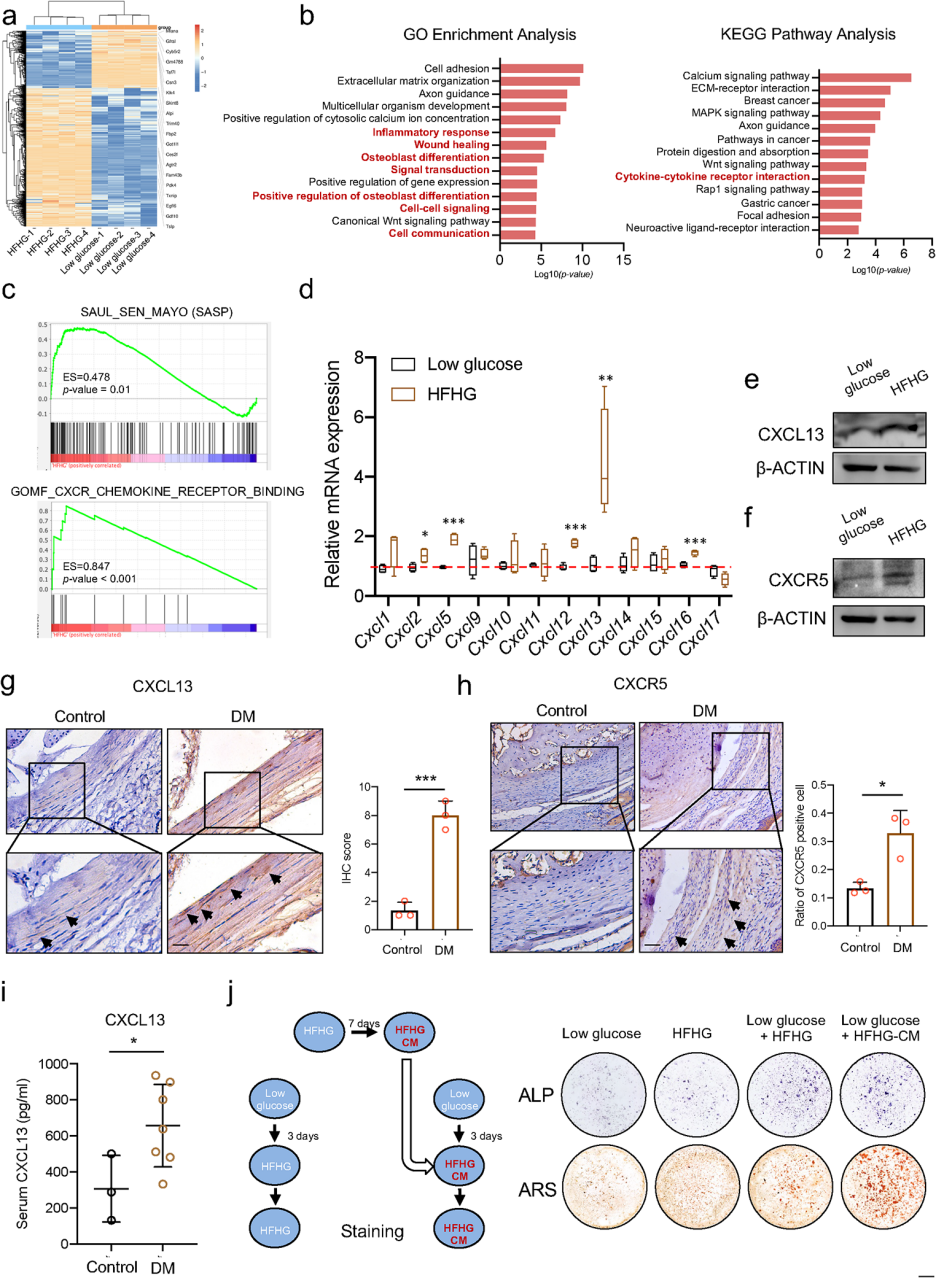
Increased levels of CXCL13 in DM mice promote the osteogenesis of TSCs. **a**. mRNA fold changes of differentially expressed genes of RNA extracted from TSCs cultured in HFHG and low glucose. Four biological replicates in each group. **b**. GO enrichment analysis (left) and KEGG pathway enrichment analysis (right) of upregulated genes (log_2_FC > 1, *p-*value < 0.05) according to RNA-seq results. **c**. GSEA analysis using SASP and CXCR genesets. **d**. The expression of mRNA of each C-X-C ligand of TSCs cultured in low glucose medium and HFHG medium. The red dotted line indicates the 1-fold. **e**–**f**. Protein levels of CXCL13 (e) and CXCR5 (f) of TSCs cultured in low glucose medium and HFHG medium. **g**–**h**. CXCL13 (g) and CXCR5 (h) staining of the Achilles tendons of Control mice and DM mice and their quantification. Scale bar = 100 μm. **i**. The level of CXCL13 in the serum of the Control and DM mice. **j**. Diagram of production of HFHG-CM and the ALP staining and alizarin red staining of different treatments, as indicated. Scale bar = 1 mm. *The p*-value was calculated by a two-tailed unpaired Student’s t-test. *p < 0.05; **p < 0.01; ***p < 0.001

To explore how the disordered glycolipid metabolism promoted the osteogenesis of TSCs, TSCs were cultured with low glucose and HFHG for four days, and the RNA was extracted for RNA-seq. A total of 849 upregulated genes were detected in the HFHG groups compared with the low glucose groups (Fig. 4a). We subjected the upregulated genes in the HFHG groups to Gene Ontology (GO) enrichment analysis, and the terms, including inflammatory response, wound healing, and osteoblast differentiation, were significantly enriched (Fig. 4b), consistent with our former results. Interestingly, the terms about cell–cell signaling and cell communication were also enriched (Fig. 4b), suggesting that some signal molecules between cells may be responsible for the mechanism. The Kyoto Encyclopedia Gene and Genomes (KEGG) pathway enrichment indicated that cytokine–cytokine receptor interaction was significantly enriched (Fig. 4b). Gene Set Enrichment Analysis (GSEA) was then conducted to explore which kind of cytokine functions in the cell signal. We found that the gene set of SASP and CXCR_Chemokine_receptor_binding was significantly enriched in the HFHG group, suggesting that the chemokine signal changed in HFHG group (Fig. 4c). To further test which chemokine changed most significantly due to disordered glycolipid metabolism, we screened the family of chemokine C-X-C motif ligands and detected that CXCL13 was upregulated, most notably in the HFHG groups (Fig. 4d–e). Previous studies have shown that CXCL13 interacts with the C-X-C motif receptor 5 (CXCR5) in regulating homeostasis. The expression of CXCR5 was also increased in the HFHG groups (Fig. 4f). Coincident with the in vitro results, the expression of CXCL13 and CXCR5 was also upregulated in the tendons of DM mice compared to Control mice (Fig. 4g–h). In addition, the serum concentration of CXCL13 also increased in the DM mice (Fig. 4i). To confirm that CXCL13 functions in a secretion manner, we cultured TSCs using HFHG medium for seven days to produce the HFHG-conditioned medium (HFHG-CM); then, we treated the TSCs with HFHG-CM or HFHG, respectively. Enhanced osteogenesis was detected in the HFHG-CM group (Fig. 4j), suggesting that CXCL13 promoted osteogenesis via an autocrine pattern. Taken together, the above results indicated that disordered glycolipid metabolism promotes the secretion of CXCL13 and then stimulates the osteogenesis of TSCs.

### The secretion of CXCL13 was promoted by senescent TSCs

**Fig. 5:**
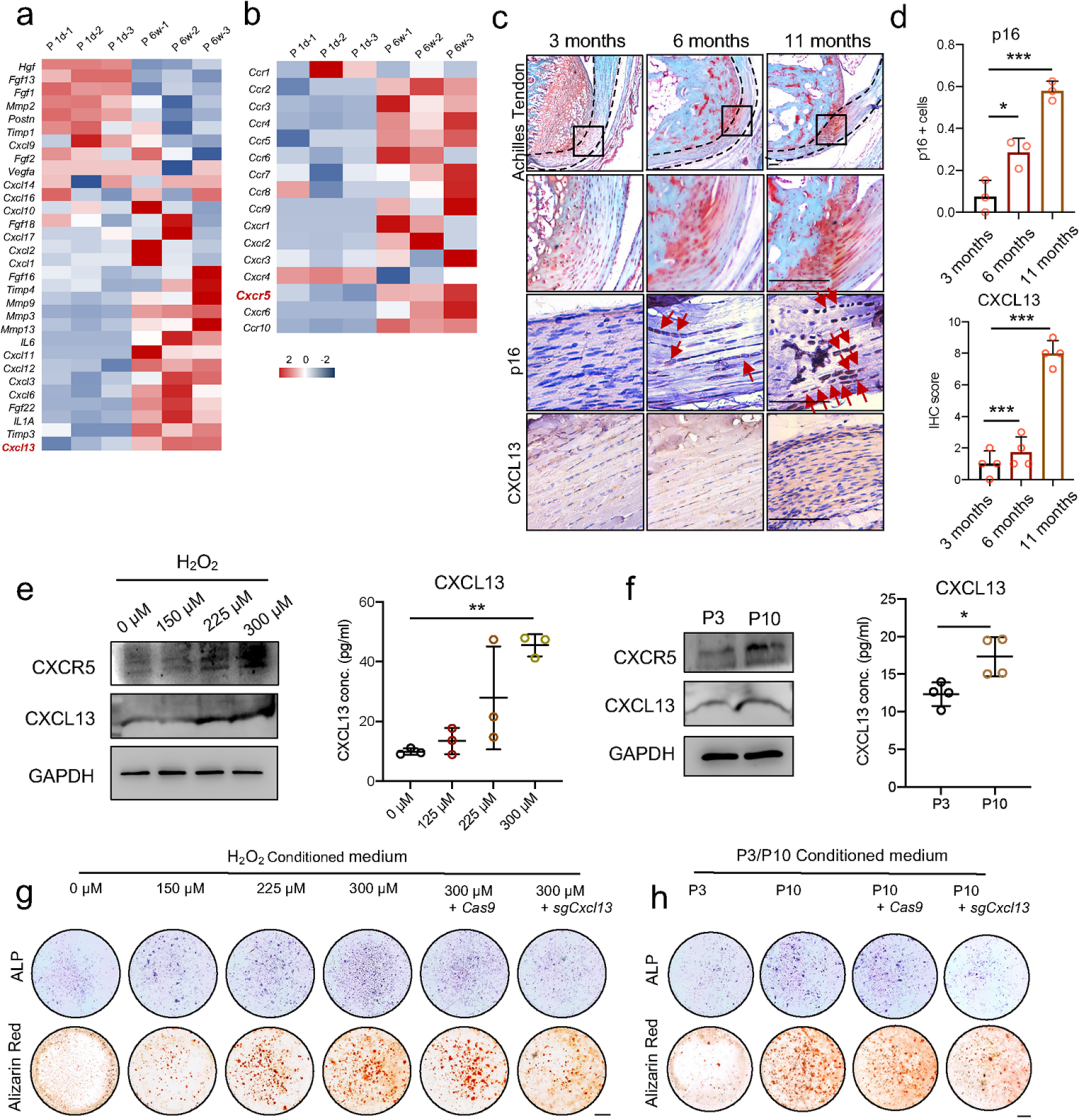
The secretion of CXCL13 was promoted by senescent TSCs. **a**–**b**. mRNA fold changes of multiple secretory factors (a) and C-X-C receptors (b) of 1-day-old and 6-week-old rat tendon tissue, according to the RNA-seq results (GSE158342). **c**–**d**. Safranine O staining, p16 staining, and CXCL13 staining in 3-month-old, 6-month-old, and 11-month-old mice Achilles tendon (c) and their quantification (d). Dashed lines indicate the Achilles tendon. Red arrows indicate the p16-positive cells. Scale bar = 100 μm. **e**. Protein levels of CXCR5 and CXCL13 and CXCL13 concentrations of TSCs cultured in 0, 150, 225, or 300 µM H_2_O_2_. **f**. Protein levels of CXCR5 and CXCL13 and CXCL13 concentrations of P3 TSCs and P10 TSCs. **g**. ALP staining and alizarin red staining of TSCs cultured in 0, 150, 225, or 300 µM H_2_O_2_ and knocked down by *Cxcl13* using CRISPR/Cas9. Scale bar = 1 mm. **h**. ALP staining and alizarin red staining of P3 TSCs and P10 TSCs and being knocked down by *Cxcl13* using CRISPR/Cas9. Scale bar = 1 mm. The *p*-value was calculated by one-way ANOVA followed by Tukey’s multiple comparisons tests (d-e) or a two-tailed unpaired Student’s t-test (f). Data are shown as mean ± SD. *p < 0.05; **p < 0.01; ***p < 0.001

In order to confirm the hypothesis that CXCL13 was induced by senescent TSCs, aged mice were used to detect the functional change of tendons. First, we took advantage of the RNA-seq dataset using rat tendons at the ages of 1 day (P 1d) and 6 weeks (P 6w) (GSE158342). We found that the expression of CXCL13, as well as its receptor CXCR5, increased significantly at P 6w (Fig. 5a–b). Then, the Achilles tendons of mice at the ages of 3 months, 6 months, and 11 months were isolated, and stronger osteochondrogenic changes were detected at 11 months and 6 months compared to 3 months (Fig. 5c). The p16-positive cells and the expression of CXCL13 also showed an increasing trend from 3-month-old mice to 11-month-old mice (Fig. 5c–d). To imitate senescence, two different kinds of in vitro models were also used, as mentioned above. The expression of CXCL13 and CXCR5 was upregulated in both senescent models, including those treated with a high concentration of H_2_O_2_ and passaged to P10 (Fig. 5e–f). The concentration of CXCL13 in the culture medium also increased (Fig. 5e–f), suggesting that senescent cells promoted the secretion of CXCL13. Similar to the result of HFHG-CM, by treating TSCs with conditioned medium from H_2_O_2_ or P10 senescent models, a significantly increased osteogenesis of TSCs was detected (Fig. 5g–h). In contrast, we knocked out *Cxcl13* of TSCs using CRISPR/Cas9 before the senescent inducement, and the enhanced osteogenesis induced by the conditioned medium was partial rescued (Fig. 5g–h). Thus, the above data suggest that elevated levels of CXCL13 increase the osteogenesis of senescent TSCs.

### Suppressing CXCL13 alleviated TSC osteogenesis and HO formation, both in vitro and in vivo

**Fig. 6:**
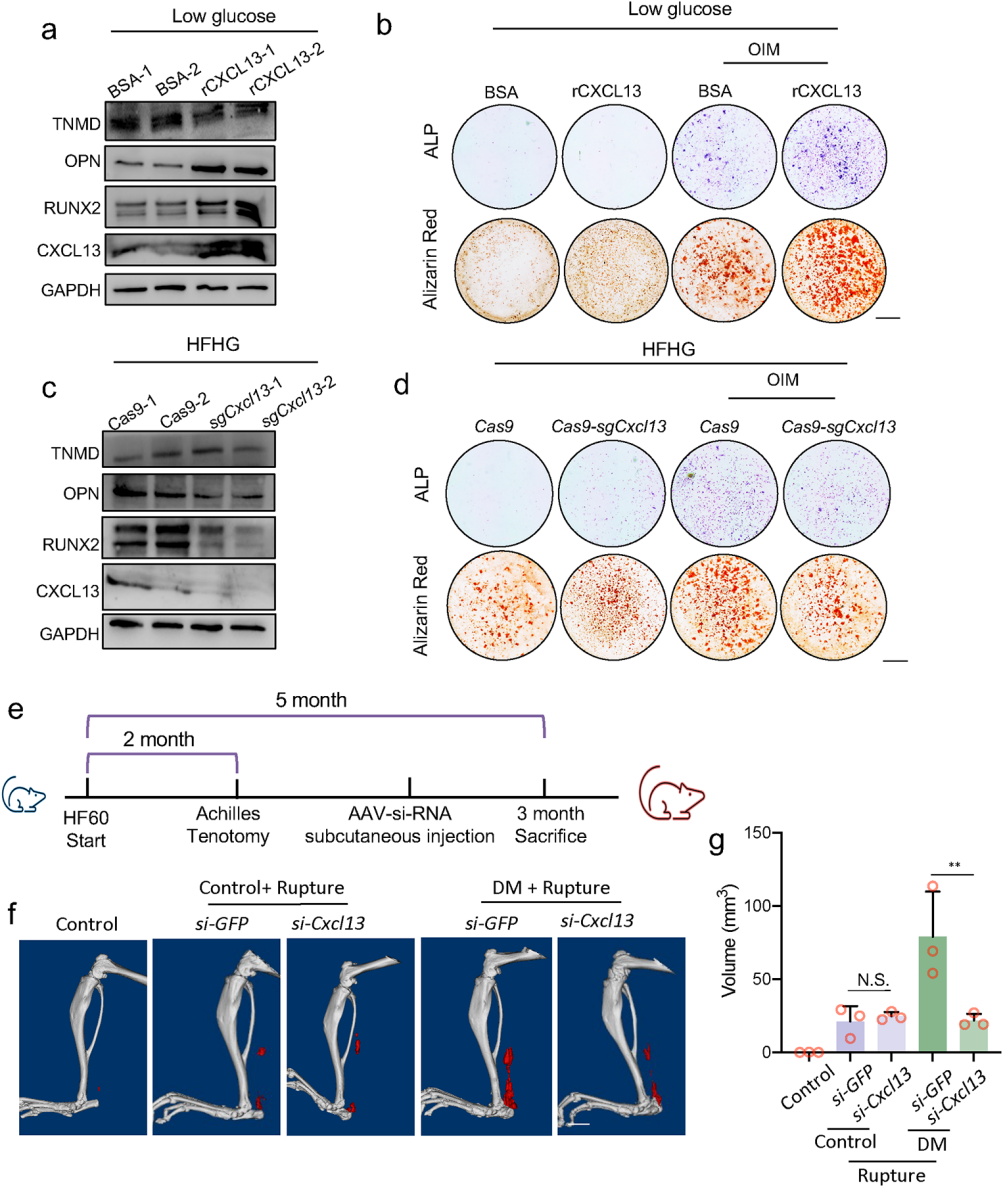
Suppressing CXCL13 alleviated TSC osteogenesis and HO formation, both in vitro and in vivo. **a**. Protein levels of TNMD, OPN, RUNX2, and CXCL13 of TSCs cultured in low glucose medium treated with BSA or recombinant CXCL13 (rCXCL13). **b**. ALP staining and alizarin red staining of TSCs cultured in low glucose medium and treated with BSA or rCXCL13 under control or OIM conditions. Scale bar = 1 mm. **c**. Protein levels of TNMD, OPN, RUNX2, and CXCL13 of TSCs cultured in HFHG medium infected with *Cas9* or *sgCxcl13* lentivirus. **d**. ALP staining and alizarin red staining of TSCs cultured in HFHG medium and infected with *Cas9* or *sgCxcl13* lentivirus under control or OIM conditions. Scale bar = 1 mm. **e**. Diagram of the construction of DM mice and treatment with tenotomy and AAV injections. **f**-**g**. CT scanning of control or DM mice injected with *AAV-si-GFP* or *AAV-si-Cxcl13* (f) and the quantification of the volume of ectopic bone (g). The *p-*value was calculated by one-way ANOVA, followed by Tukey’s multiple comparisons tests. Data are shown as mean ± SD. **p < 0.01. N.S., not significant

Previous studies showed that CXCL13 mediated the immunoreaction of multiple immune cells, but there was still no evidence of whether CXCL13 regulated osteogenesis. To illustrate the function of CXCL13 during TSC osteogenesis, we treated TSCs with exogenous recombined CXCL13 (rCXCL13). Decreased tenogenesis markers and increased osteogenesis markers were detected in the rCXCL13-treated group (Fig. 6a). Under OIM conditions, the osteogenesis of the TSCs was further enhanced (Fig. 6b). Conversely, when the expression of *Cxcl13* in TSCs was knocked out using CRISPR/Cas9, the decreased tenogenesis marker and increased osteogenesis marker under the HFHG condition were significantly rescued (Fig. 6c), and the enhanced osteogenesis under the OIM condition was also partially rescued (Fig. 6d). Thus, the data suggest that suppressing CXCL13 alleviates TSC osteogenesis in vitro. We then explored the therapeutic potential of suppressing CXCL13 in vivo. We used siRNA packed in adeno-associated virus 2 (AAV2) to knock out *Cxcl13* in the Control mice and DM mice after exerting Achilles tenotomy (Fig. 6e). After three months, less ectopic bone formation was detected in DM mice with tendon rupture, while no significant rescue was observed in Control mice (Fig. 6f–g). Thus, suppressing CXCL13 was sufficient to alleviate HO in DM mice after tendon trauma.

### Increased CXCL13 expression in human ossified ligament and DM patient blood serum

**Fig. 7:**
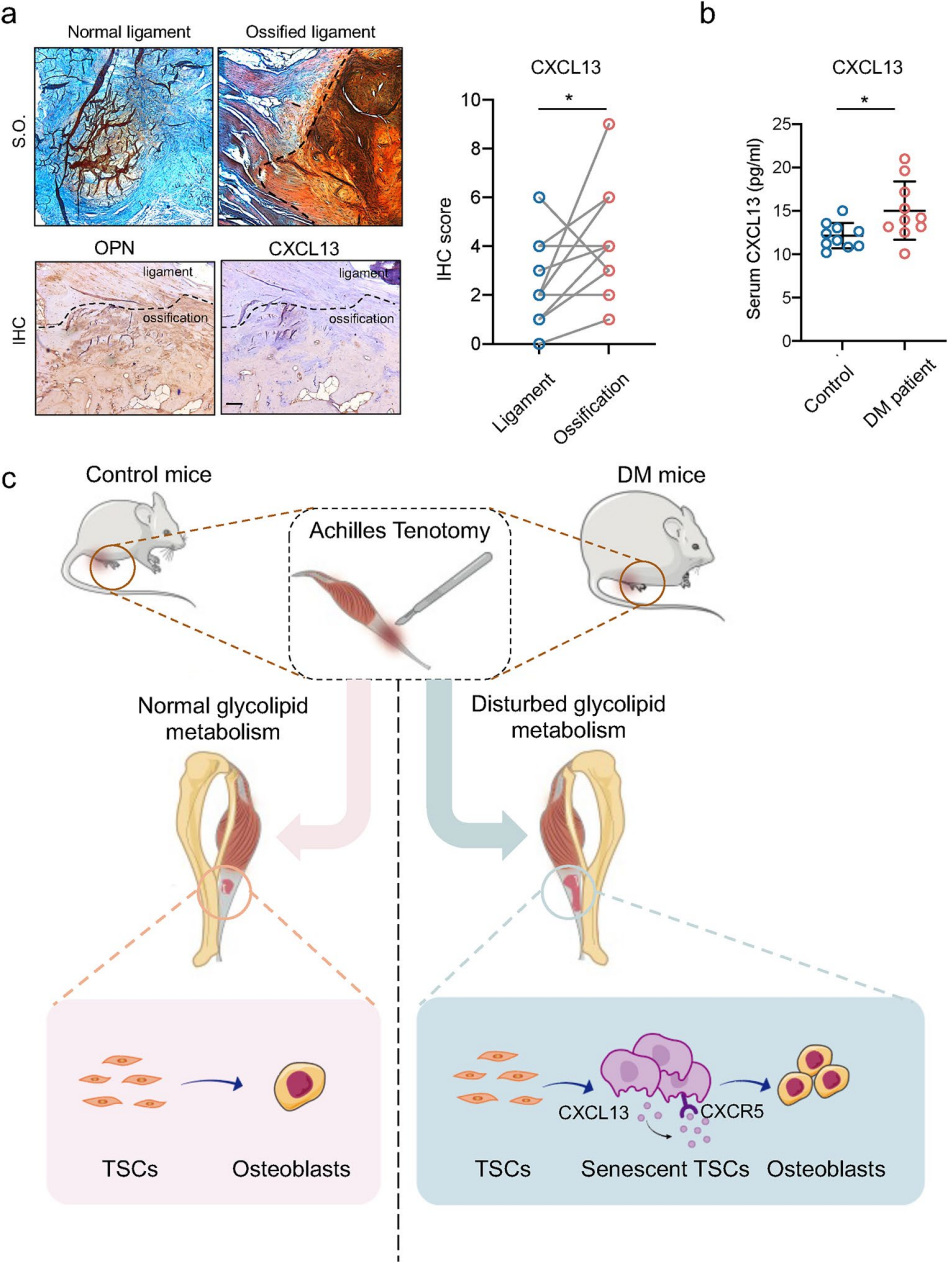
Increased CXCL13 expression in human ossified ligament and DM patient blood serum. **a**. Safranine O staining, OPN, and CXCL13 staining in normal human ligaments and ossified human ligaments and their quantification. Scale bar = 100 μm. **b**. Level of CXCL13 in the serum of DM patients. **c**. Disordered glycolipid metabolism results in the cellular senescence of TSCs. The senescent TSCs activated the CXCL13-CXCR5 axis in an autocrine pattern and enhanced the osteogenesis of the TSCs. The *p*-value was calculated by paired Student’s t-test (a) or two-tailed unpaired Student’s t-test (b). Data are shown as mean ± SD. *p < 0.05

We isolated the ossified ligament from patients who underwent spine surgery, and normal ligaments and ossification were observed using Safranine O staining (Fig. 7a, upper panel). A stronger expression of OPN and CXCL13 was observed in the ossified region than in the ligament region (Fig. 7a). Finally, we measured the blood serum concentration of CXCL13 in normal humans and DM patients, and an increased concentration of CXCL13 was detected in DM patients (Fig. 7b). Thus, the data indicated that an increased CXCL13 level was one of the risk factors of developing ligament HO in DM patients.

## Discussion

The risk factors and pathological mechanisms of HO have not yet been totally illustrated due to the multiple sources of cell subsets. In this study (Fig. 7c), we reported enhanced ectopic bone formation occurring in the tendons of patients or mice suffering from DM after trauma. Mechanically, we identified that disordered glycolipid metabolism aggravated TSC senescence and activated the CXCL13-CXCR5 axis in TSCs, which led to enhanced osteogenic differentiation of TSCs.

CXCL13 is one of the members of the C-X-C motif ligand family, mostly secreted from macrophages, and it is essential for B-cell trafficking and monocyte activation. CXCR5 was the specific receptor of CXCL13, and the CXCL13-CXCR5 axis was proved to be activated in carcinogenesis, development, and metastasis in various malignant tumors and differentiation of immune cells [20–22]. Patients with carotid atherosclerosis showed increased plasma levels of CXCL13, where CXCL13 exerted anti-apoptotic effects and counteracted the suppressive effects of IL-1b on collagen synthesis in smooth muscle cells (SMCs) [23]. CXCL13 has also been suggested to play a pathogenic role in inflammatory disorders, such as rheumatoid arthritis and inflammatory bowel disease [24, 25]. However, the function of CXCL13 has not been previously reported in TSCs. In the present study, increased levels of CXCL13 were detected in both HFHG cells and the DM mice model. Further experiments revealed that disordered glycolipid metabolism promoted the secretion of CXCL13 and stimulated the osteogenesis of senescent TSCs. Similarly, another member of the C-X-C motif ligand family, CXCL12, which was expressed in mesenchymal cells, was capable of inducing MSC migration and osteogenesis in OLF, and its expression in macrophages increased new bone formation in ankylosing spondylitis. Taken together, the current study revealed that CXCL13 was not only expressed in mesenchymal cells like TSCs but also regulated the proliferation and differentiation of TSCs in an autocrine pattern.

Previous reports identified that the cells which participated in the occurrence of HO were from tissue-resident mesenchymal, vascular, circulating, hematopoietic, and bone marrow derived populations [26–30]. In tendon HO, TSCs were recognized as the responsible cell population, and our in vitro experiments confirmed that TSCs cultured in HFHG were prone to osteoblastic differentiation. Using an extreme HO model, fibrodysplasia ossificans progressiva (FOP), the origins of the cell population were identified as *Scx+* tendon-derived progenitor mediating endochondral HO and *Mx1+* muscle-resident interstitial population mediating intramuscular HO [30]. Similar to the bone formation in FOP, the pattern of trauma-induced HO in our study was also endochondral ossification [31]. As our data showed, the Achilles tendons of aged mice and DM mice, especially the junction between tendon and articular cartilage, exhibited chondrogenic changes, which indicated the progress of endochondral ossification. Thus, disordered glycolipid metabolism promotes ectopic bone formation through endochondral ossification.

DM influenced multiple widespread systems and was one of the stresses accelerating cellular senescence [32]. Cellular senescence is a stable cell cycle arrest and senescence-associated secretory phenotype (SASP) characterized by the upregulation of pro-inflammatory factors. Senescent progenitor cells exhibit changes in differentiation capacity or aberrant lineage distribution [33–35]. For example, in a high-fat, diet-induced diabetes mice model, the adipose tissue displayed an increased accumulation of *p21*^*cip1*^-highly-expressing and *p16*^*INK4a*^-highly-expressing cells [36]. In our study, we also discovered that the TSCs from DM mice exhibited significant cellular senescence and SASP, suggesting that cellular senescence was the transitional stage of HO. The former study discovered decreased stemness and proliferative capacity in aged bone marrow-derived stem cells (BMSCs) and aged TSCs [37, 38], while the research of osteogenesis of aged TSCs was lacking. Recently, research showed that, during wound healing, pyroptotic macrophages aroused TSCs senescence and encouraged HO formation after trauma [35]. As well as an acquired form of HO, the bone morphogenetic protein (BMP) signal inducer activin A, which was the trigger of FOP [39], was a SASP [40]. Thus, cellular senescence might play an important role in HO development. In our study, we used two in vitro senescent models [41, 42] and aged mice to illustrate that the senescent TSCs exhibited higher osteogenic potential, contributing to the occurrence of HO. In other words, cellular senescence significantly changed the differentiation tendency of TSCs. Due to the function of cellular senescence in HO, senotherapeutic agents could be potential drugs for the prevention and treatment of HO. Senescent cell accumulation was found in the muscle injury FOP model, promoting tissue reprogramming toward a chondrogenic fate in the FOP muscle, and the senolytic drug successfully ameliorated the FOP in mice [43]. Thus, the therapeutic potential of anti-senescence in HO formation is worthy of further study.

Until now, there have been no effective diagnoses or treatment options for HO. Molecular biomarkers, such as MCP-1 and IL-1β, were reported to be related to the progression of HO [44]. Traditional first-line medications such as NSAIDs and corticosteroids are used in an attempt to prevent HO but are often ineffective, indicating the need for new prophylactic measures that can be a viable option for HO patients [45, 46]. In recent studies, new prophylactic drugs such as Saracatinib or Palovarotene were tested as potential treatment in FOP-related clinical trials [47, 48]. In this study, we offered a potential therapeutic target for DM patients by using CXCL13-neutralizing antibodies after trauma. The CXCL13 neutralizing antibody has been reported to be used in hyperalgesia [20], pancreatic ductal adenocarcinoma [49], and renal cell carcinoma [22]. In our study, we used AAV2 to knock down the expression of CXCL13 in tendons because the affinity of AAV2 was proved in tendons, according to the former report [50]. We did not observe any signs of severe side effects during or after treatment with AAV2. Compared to using AAV for clinical applications, the neutralizing antibody was safer and easier to use. For example, the neutralizing antibody for RANKL, denosumab, exhibited benefits for osteoporosis patients and giant-cell tumors of bone patients [51, 52]. Thus, using CXCL13-neutralizing antibodies to prevent HO in DM patients after surgery or trauma could be a new orientation of therapy.

In order to distinguish whether the elevated secretion of CXCL13 was the reason or the result of senescent TSCs, we knocked out CXCL13 in TSCs using CRISPR/Cas9, and the cellular senescence induced by H_2_O_2_ was alleviated. Interestingly, adding the recombinant protein CXCL13 did not induce cellular senescence (Supplementary Fig. S5A-B), suggesting that the elevated CXCL13 level was the result of cellular senescence but not the reason. Further research about the cause of disordered glycolipid metabolism inducing TSC senescence is still needed.

There are still some limitations to our study. First, we used AAV to inhibit CXCL13 in the tendon, which was not specifically enough for TSCs. Because the local macrophage also secreted CXCL13, we could not eliminate that part of CXCL13 originating from immune cells. The application of *Scx-cre* transgenic mice for CXCL13 specific ablation in TSCs was helpful to illustrate the conclusion [53]. Second, the number of clinical samples was insufficient and from one center, which may have caused selection bias. Follow-up, multiple-center validation could offer stronger evidence for the conclusion. Finally, though CXCL13 AAV2 exhibited a preliminary effect in the treatment of HO in mice, more in vivo studies are required to define the efficacy, toxicity, window of treatment, duration of exposure, and dosing interval of CXCL13.

## Materials and methods

### Human subjects

This study was approved by the Medical Ethics Committee of Sun Yat-sen Memorial Hospital of Sun Yat-sen University (SYSKY-2023-342-01), and signed informed consent forms were obtained from each subject. We collected 60 patients who underwent surgery at Sun Yat-sen Memorial Hospital of Sun Yat-sen University due to cervical spondylosis and divided them into two groups: the Control group (*n* = 40) and the Hyperglycemia group (*n* = 20). We analyzed their preoperative CT scanning data and calculated the rate of HO that occurred in the ligamentum nuchae (OLN), posterior longitudinal ligament (OPLL), or ligamentum flavum (OLF). The calcified tendon was obtained from 12 patients with HO during surgery. We also collected the normal Achilles tendon from patients who received surgery (*n* = 5) and from DM patients who received surgery due to the diabetic foot (*n* = 5). The serums of the control patients (*n* = 10) and DM patients (*n* = 10) were collected for the ELISA test.

### Mice

All mice (C57BL/6J) were purchased and bred at the Laboratory Animal Center of Sun Yat-sen University. The animal experiments were approved by the Institutional Animal Care and Use Committee of Sun Yat-Sen University (SYSU-IACUC-2023-000847). We used mice aged from 6 to 8 weeks for the DM model by feeding them with 60% high-fat fodder (HF60 fodder, Dyets) for two months and then exerted Achilles tenotomy to induce the occurrence of HO. We measured the blood glucose level using a glucometer, and a blood glucose higher than 33.3 mmol/L was displayed as High, and lower than 1.1 mmol/L as Low. Before the glucose tolerance test (GTT) and insulin tolerance test (ITT), the mice fasted for eight hours. For the GTT, we treated the mice with 2 g/kg glucose via intraperitoneal injection and the insulin level was measured using ELISA (E-EL-M2614c-96T, Elabscience). For ITT, we treated the mice with 1 U/kg insulin via intraperitoneal injection.

### Histological analysis

The tissues were disserted and fixed in 4% paraformaldehyde for 48 h at 4 °C. After fixation, the tissues were embedded in paraffin and processed for paraffin sectioning at 5 μm thickness. H&E staining, Safranine O staining, and Masson trichrome staining were performed according to standard procedures. The slides were photographed under a light microscope (Olympus).

### Immunohistochemistry

The slides were immunoreacted with anti-TMND (Abcam, ab203676, 1:250), anti-RUNX2 (Abcam, ab192256, 1:500), anti-OPN (Abcam, ab214050, 1:500), anti-p16 (Santa Cruz, sc-1661, 1:100), and anti-CXCL13 (Abcam, ab272874, 1:100) or anti-CXCR5 (Abcam, ab203212, 1:500). Further processing occurred according to standard procedures. The IHC score was calculated independently by experimenters blinded to the sample identity. Staining intensity was scored as follows: 0 (negative), 1 (weakly positive), 2 (moderately positive), and 3 (strongly positive). The percentage of positivity was also scored according to five categories: 0 (< 5%), 1 (5–25%), 2 (25–50%), 3 (50–75%), and 4 (> 75%). The value of the percentage positive score was multiplied by the staining intensity score to generate the final expression scores, which ranged from 0 to 12.

### Isolation and culture of TSCs

The patellar tendon was isolated and washed using PBS, then cut into small pieces and digested with type I collagenase (3 mg/ml, Sigma-Aldrich) for 6 h at 37℃. The cells were then resuspended and seeded in a 6-well plate and cultured at 37 °C with 5% CO_2_. Low glucose DMEM (Gibco) supplemented with 1% penicillin–streptomycin solution (Gibco) and 10% fetal bovine serum (Gibco) were used as the culture medium. For osteogenic induction, the OIM was the culture medium supplemented with 20 mM β-glycerolphosphate, 50 mM ascorbic acid, and 1 nM dexamethasone for 7 or 14 days. The alkaline phosphatase (ALP) staining and alizarin red staining were performed according to standard procedures.

### Cell Counting Kit-8 assay (CCK-8)

The TSCs were seeded at a density of 1 × 10^4^ per well in 96-well plates and cultured in culture medium or high glucose DMEM supplemented with different concentrations of palmitic acid for 0, 12, 24, and 48 h. CCK-8 solution (Glpbio) was added to each well and incubated for 3 h, followed by measurement of the absorbance at 450 nm using a microplate reader (TECAN sunrise).

### Immunoblotting analysis

We performed western blotting according to standard procedures. The following primary antibodies were used: anti-OCT4 (CST, #2750, 1:500), anti-NANOG (Abcam, ab109250, 1:500), anti-p21 (Abcam, ab109520, 1:1,000), anti-TNMD (Abcam, ab203676, 1:250), anti-RUNX2 (Abcam, ab192256, 1:1,000), anti-OPN (Abcam, ab214050, 1:500), anti-Cyclin D1(Santa Cruz, sc-8396, 1:500), anti-Cyclin B1 (Santa Cruz, sc-245, 1:500), anti-CXCL13 (Abcam, ab272874, 1:250), anti-CXCR5 (Abcam, ab203212, 1:500), anti-GAPDH (Proteintech, 60004-1, 1:2,000), or anti-β-ACTIN (Affinity, AF7018, 1:2,000). The secondary antibodies used were goat anti-rabbit IgG H&L (Abcam ab205718, 1:2,000) and goat anti-mouse IgG H&L (Abcam ab205719, 1:2,000). The original blot results are shown in Supplementary Figure S6.

### Senescence-associated β-galactosidase (SA-β-gal) staining

SA-β-gal staining (Beyotime) was performed according to the manufacturer’s protocol. Briefly, the cells were washed with PBS and fixed in the SA-β-gal fixative for 15 min at room temperature, followed by incubation with SA-β-gal working solution at 37℃ overnight. After staining, three random fields of each group were imaged under an optical microscope, and the ratio of β-Gal + cells was calculated.

### qPCR

Total RNA was extracted from cultured cells using TRIZOL reagent (Invitrogen), and cDNA was synthesized using 1,000 ng of total RNA with a Prime-Script RT reagent kit (TaKaRa) according to the manufacturer’s protocol. qPCR was performed to amplify the cDNA on a Light Cycler 480 Real-Time PCR system (Roche Light Cycler 480) using TB Green Premix Taq II (TaKaRa) and corresponding primers. The sequences of primers were acquired from PrimerBank [54], and the expected size of the amplicon in bp, the annealing temperatures, GenBank Accession numbers are listed in Supplementary Table 2. The 2^−ΔΔCt^ method was used to calculate the relative expression levels, and the expression of *Actb* served as the internal control for normalization.

### RNA-seq and analysis

Total RNA was extracted from TSCs cultured in low glucose or high fat, high glucose (HFHG), and libraries were generated using the VAHTS Stranded mRNA-seq Library Prep Kit for Illumina (New England Biolabs). Library quality was checked with a Bioptic Qsep100 Analyzer (Bioptic Inc.), and libraries were sequenced using NovaSeq 6000. Genes with a fold change > 2 and *p-*value < 0.05 were identified as differentially expressed genes and analyzed using DAVID Bioinformatics Resources. Four biological replicates in each group were included in the RNA-seq experiment in this study.

### Bone CT scanning

The hindlimbs of the mice were dissected and fixed in 4% paraformaldehyde for 48 h at 4 °C, followed by storage in 70% ethanol for micro-CT scanning (SCANCO Medical AG). Quantitative volumetric measurements of HO were conducted on the tibia region.

### CRISPR/sgRNA plasmid construction

The lenti-CRISPR v2 plasmid was available from Addgene (52,961). The targeted sgRNAs were inserted into the plasmid using BsmBI sites. The sgRNA sequences for *Cxcl13* were as follows: sgRNA-1 (TCGGTCTAAACATCATAGAT, PAM: CGG) and sgRNA-2 (GGATTCAAGTTACGCCCCCT, PAM: GGG).

### Vector transduction and lentivirus/adenovirus infection

Lentivirus infection was performed according to the manufacturer’s protocol of the GeneCopoeia Lenti-Pac kit. Briefly, HEK293T cells were plated in high-glucose DMEM (Gibco) with 10% FBS and 1% penicillin–streptomycin. The cells were co-transfected with 2.5 µg lenti-CRISPR plasmid and packing plasmids (pLP1, pLP2, and pLP-vsvg) using Lipofectamine 3000 (Thermo Fisher Scientific). After 8–14 h, the transfection medium was changed, and the medium with lentivirus was harvested after 48 h. The lentiviral supernatant was filtered through 0.45 μm filters to eliminate residual cells and cell fragments.

After the cells were attached to the dish with 70–80% confluency, the adenovirus or lentivirus was added to the non-FBS medium (10^9^ PFU/ml). The same volume medium with 10% FBS was added to the cells, and the medium was changed after 24 h. The cells were cultured for another 24 h before the next step of the experiments.

### Statistical analysis

Quantification was performed in at least three independent experimental groups. The analysis of all statistics was carried out using SPSS v13. We tested the values with the Kolmogorov–Smirnov test (K–S test) to determine the normally distributed, and the two-tailed Student’s *t*-test (normally distributed) or Mann–Whitney U test (not normally distributed) was used between two groups to determine the significance. A one-way ANOVA (normally distributed) with Tukey’s post-hoc test was used to compare differences between multiple groups. In all cases, a *p*-value of less than 0.05 was considered significant, and the results were presented as mean ± standard deviation (SD).

### Electronic supplementary material

Below is the link to the electronic supplementary material.

Supplementary Material 1

## Data Availability

The raw sequencing data were uploaded to the GEO database (GSE234916).
