# Supplementary material for: Disturbed glycolipid metabolism activates CXCL13-CXCR5 axis in senescent TSCs to promote heterotopic ossification
Source: Cell Mol Life Sci. 2024 Jun 17;81(1):265. doi: 10.1007/s00018-024-05302-3 (PMC11335191; doi:10.1007/s00018-024-05302-3)
Supplement: Supplementary file 1 — Supplementary Material 1 [file 18_2024_5302_MOESM1_ESM.docx]

**
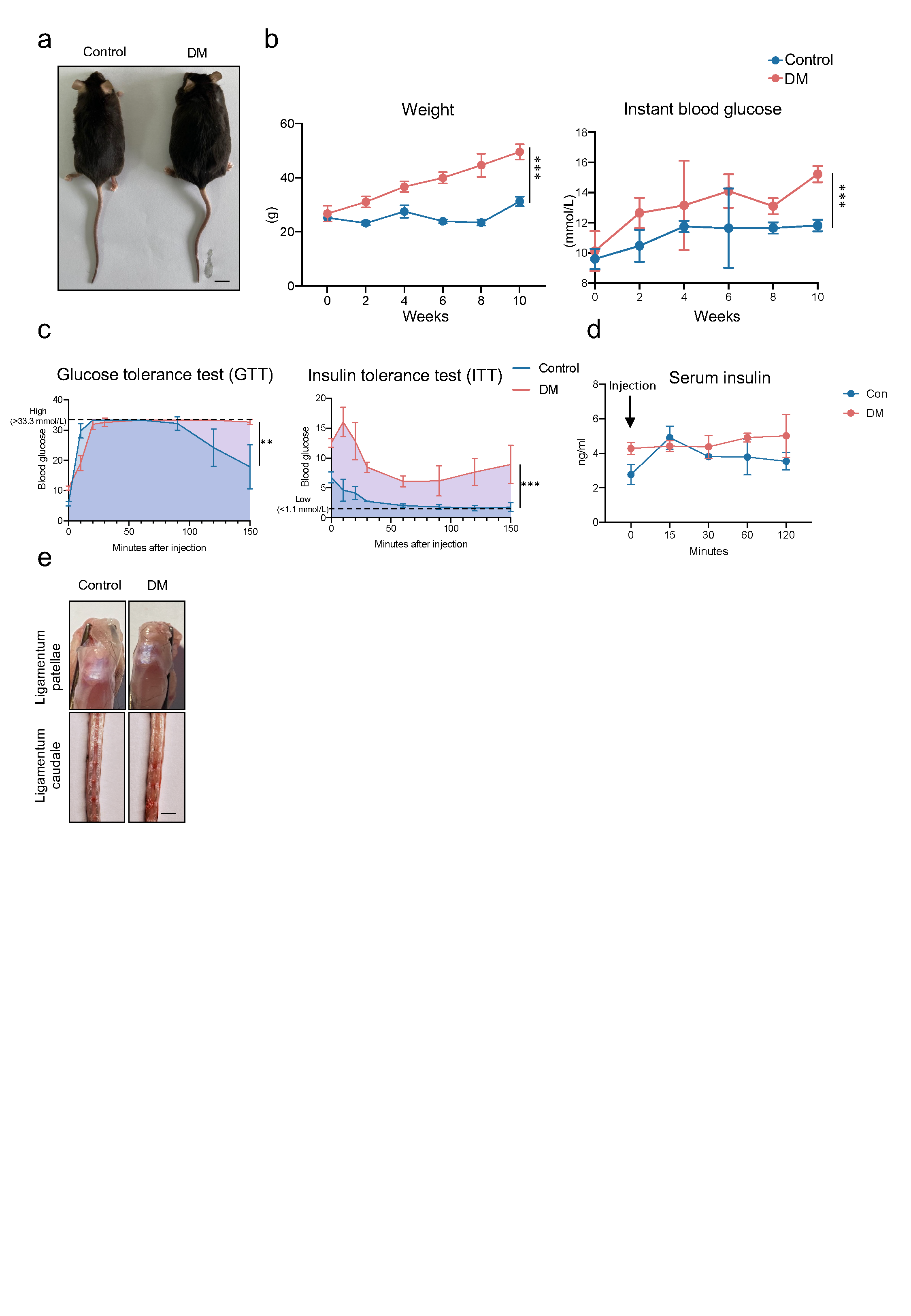
Supplementary Fig. S1 Construction of DM mice.**

a. Appearance of control mice and DM mice. Scale bar = 1 cm.

b. The weight and instant blood glucose of control mice and DM mice at week 0, 2, 4, 6, ,8 and 10 after HF60 feeding.

c. The blood glucose level of GTT and ITT of control mice and DM mice.

d. The serum insulin level of control mice and DM mice after glucose injection.

e. Appearance of ligamentum patellae and ligamentum caudale of control mice and DM mice. Scale bar = 10 mm.

*p*-value was calculated by Two-way ANOVA. Data were shown as mean ± SD. ***p* < 0.01. ****p* < 0.001.

**
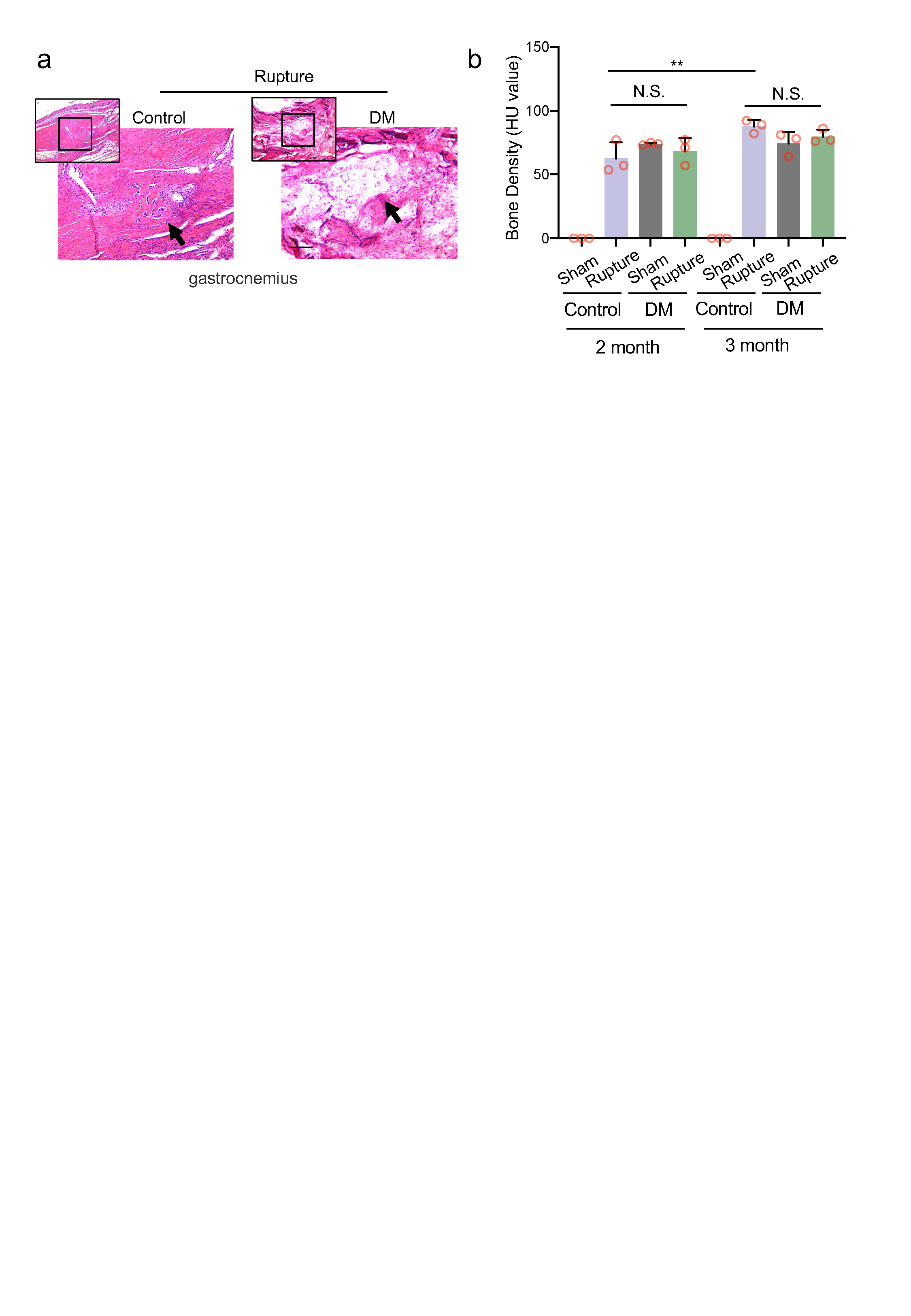
Supplementary Fig. S2 DM mice were more susceptible to developing tendon and ligament HO.**

a. H&E staining of ectopic bone formation in gastrocnemius of control mice and DM mice. Scale bar = 100 μm.

b. Bone density of ectopic bone of control mice and DM mice received sham or tenotomy for 2 or 3 months.

*p*-value was calculated by one-way ANOVA followed by Tukey’s multiple comparisons tests. Data were shown as mean ± SD. ***p* < 0.01. N.S., not significant.

**
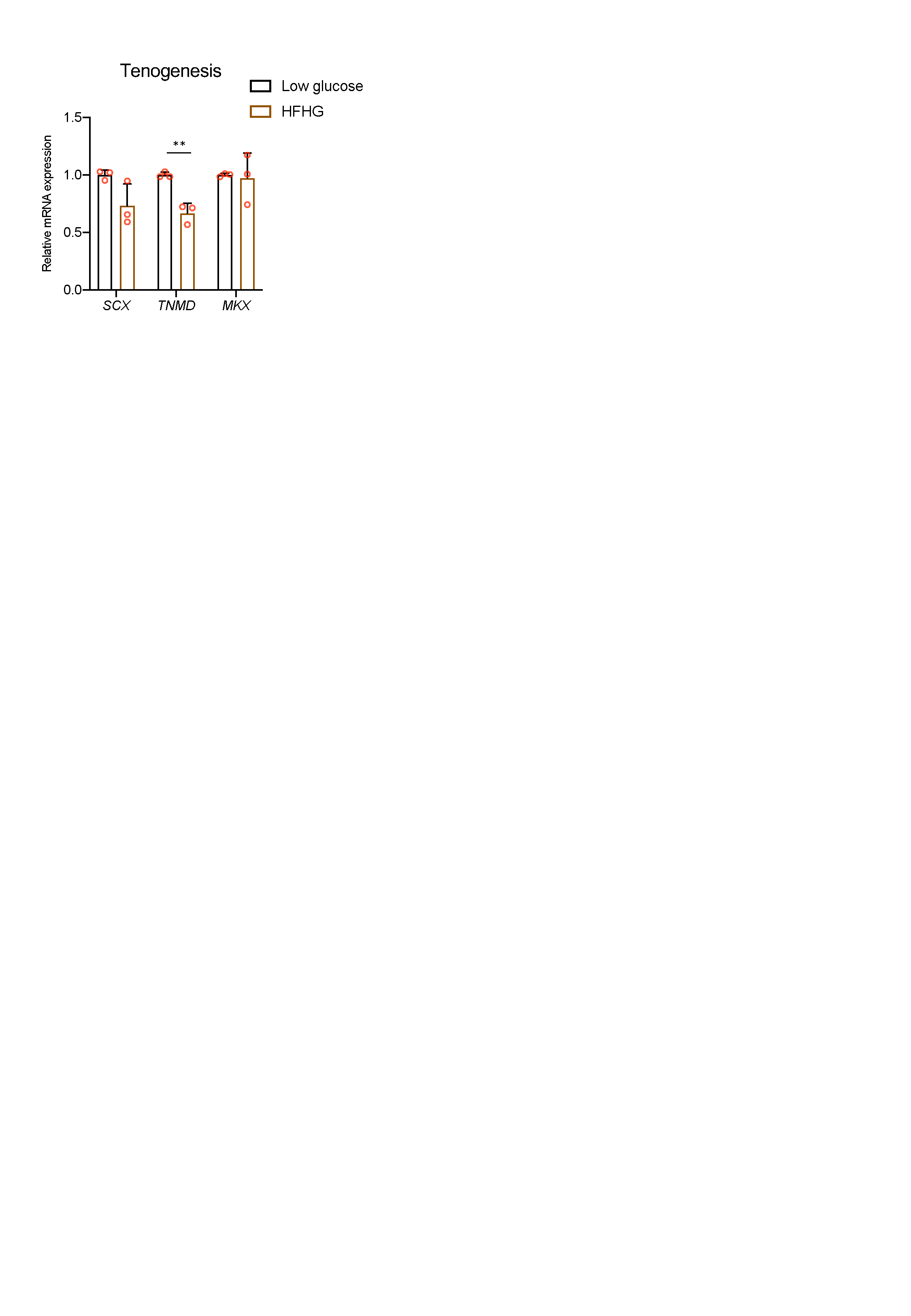
**

**Supplementary Fig. S3 Disturbed glycolipid metabolism damaged tenogenesis of TSCs.**

The expression of mRNA relevant to tenogenesis of TSCs cultured in low glucose medium and HFHG medium.

*p*-value was calculated two-tailed unpaired Student’s t-test. Data were shown as mean ± SD. ***p* < 0.01.

**
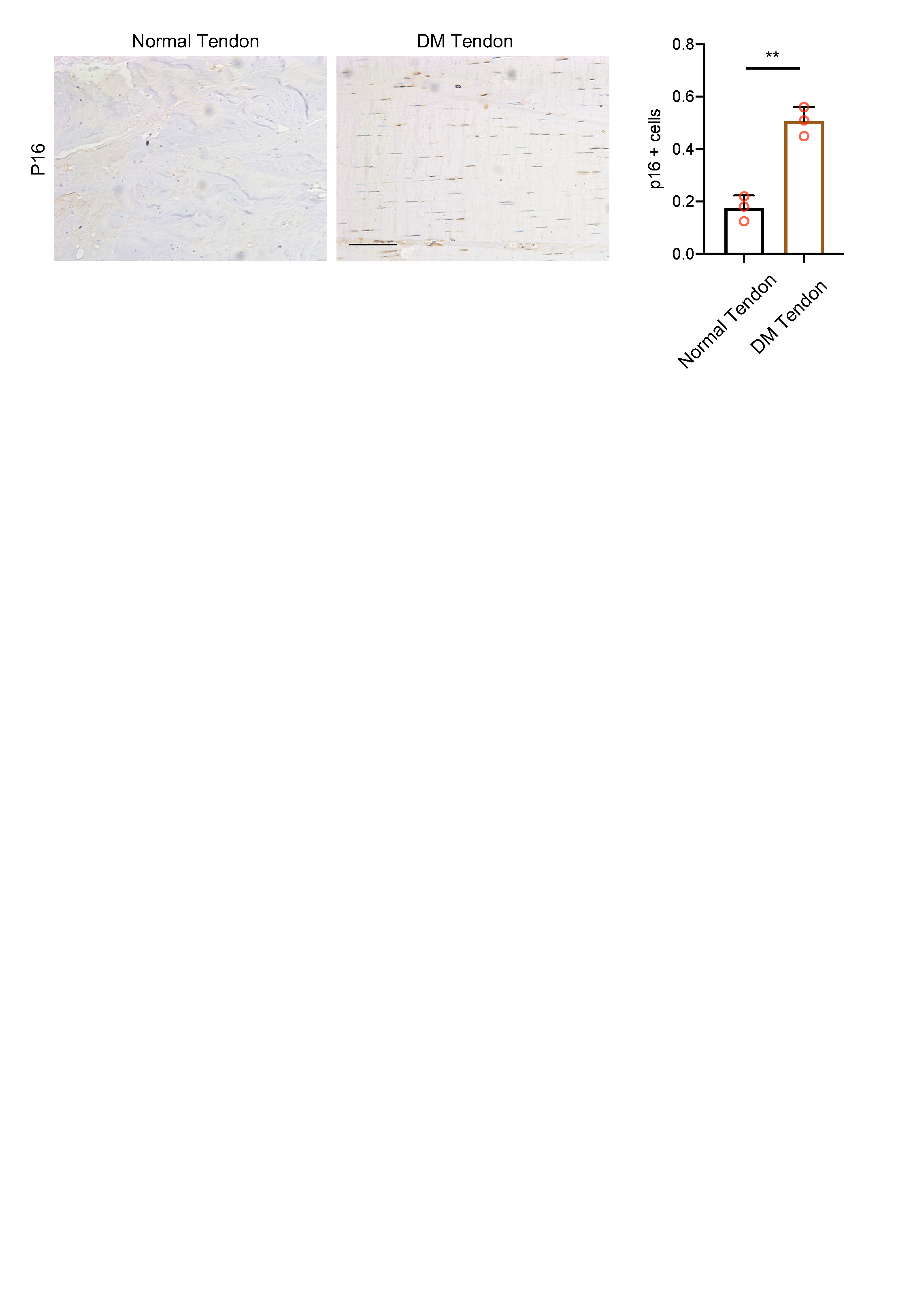
Supplementary Fig. S4 Disturbed glycolipid metabolism promoted TSCs senescence.**

The p16 staining of the Achilles tendon of normal tendon and DM tendon and their quantification. Scale bar = 100 μm.

*p*-value was calculated by two-tailed unpaired Student’s t-test. Data were shown as mean ± SD. ***p* < 0.01.


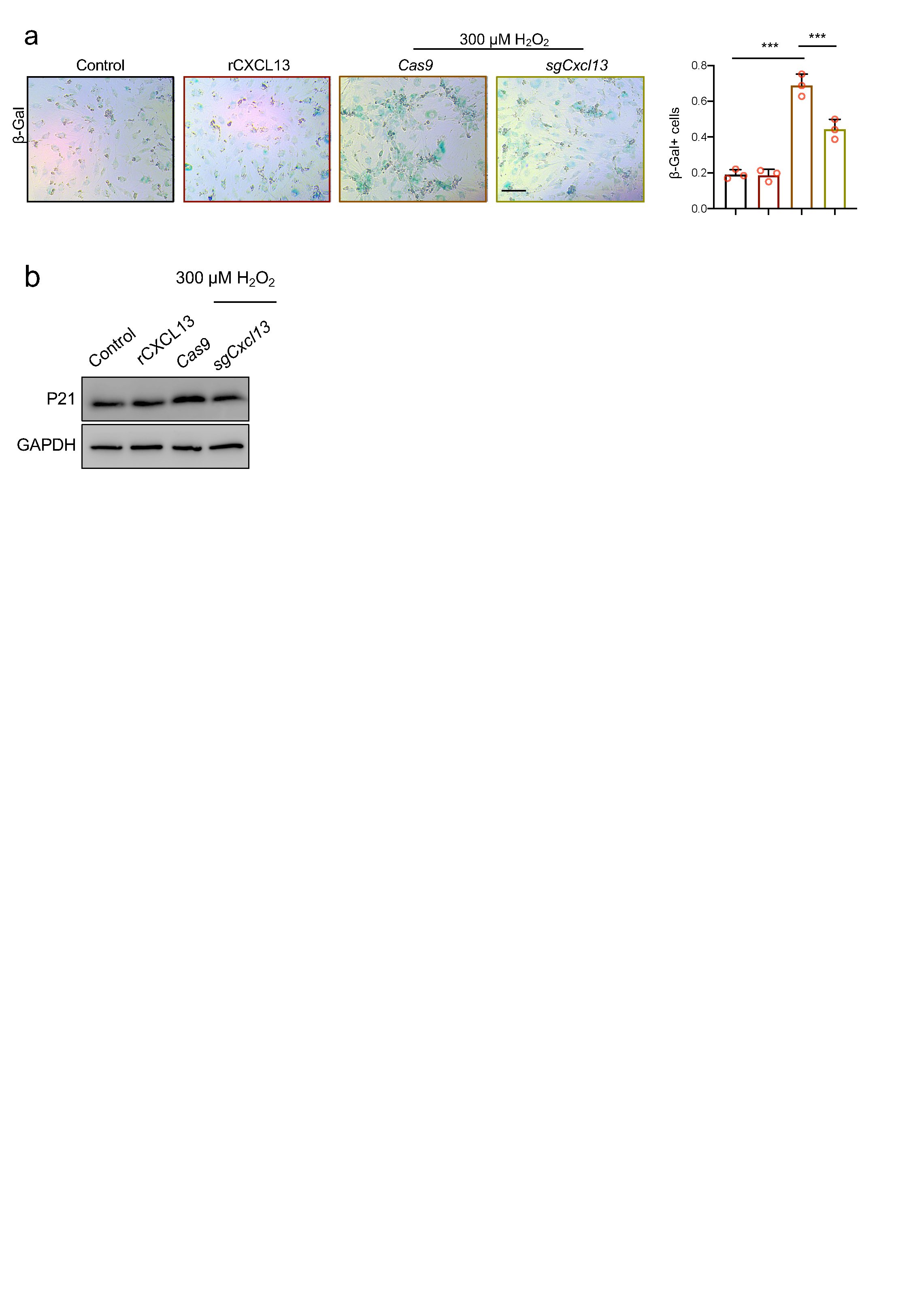


**Supplementary Fig. S5 CXCL13 was secretes by senescent TSCs.**

a. The β-Gal staining of TSCs treated with rCXCL13 or being knocked down with *Cxcl13* and their quantification. Scale bar = 50 μm.

b. Protein level of p21 of TSCs treated with rCXCL13 or being knocked down with *Cxcl13.*

*p*-value was calculated by one-way ANOVA followed by Tukey’s multiple comparisons tests. Data were shown as mean ± SD. ****p* < 0.001.

**
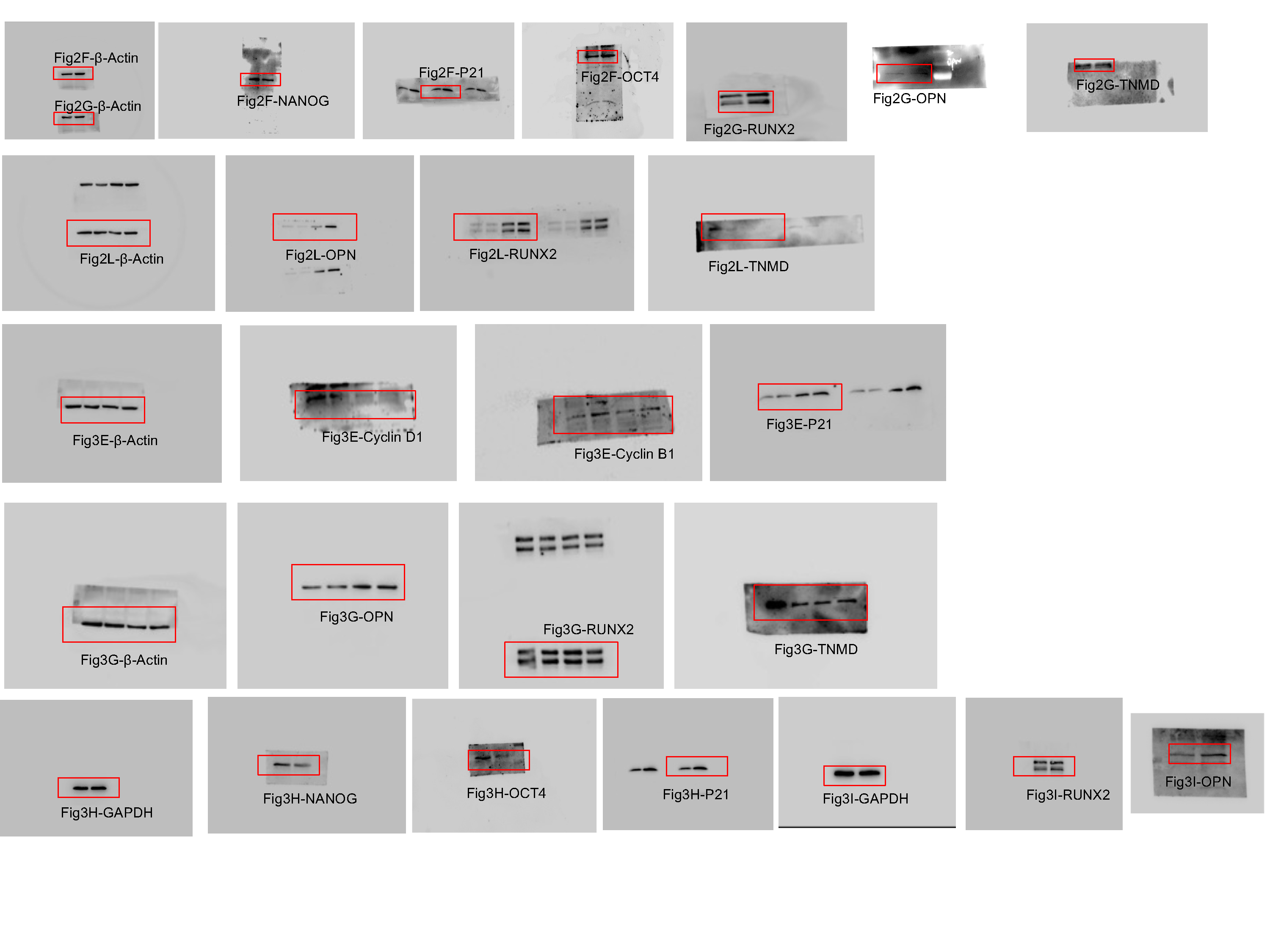
**

**
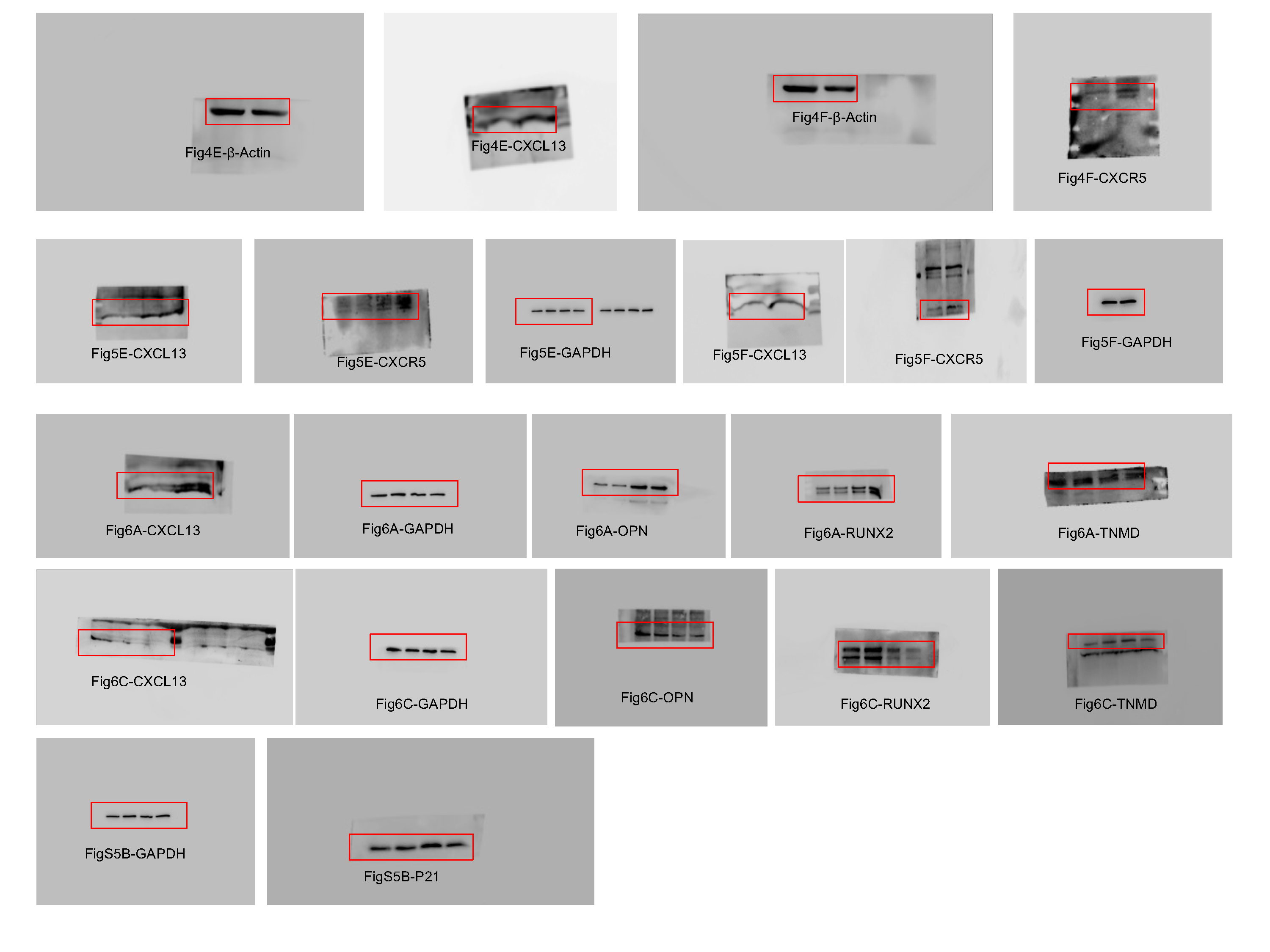
**

**Supplementary Fig. S6 Original blot data**

**Supplementary Table 1. The age, sex, BMI, and HbA1c level of healthy control patient and Hyperglycemia patient.**

|  | **Healthy control (n = 40)** | **Diabetes patients (n = 20)** | ***p-*value** |
| --- | --- | --- | --- |
| Age (years) | 59.73 ± 12.18 | 63.45 ± 8.23 | 0.226 |
| Sex, M;F (%F) | 21;19 (47.50%) | 11;9 (45%) | 0.855 |
| BMI (kg/m^2^) | 22.53 ± 2.91 | 24.02 ± 3.79 | 0.098 |
| HbA1c (%) | 4.84 ± 0.18 | 8.29 ± 1.79 | < 0.001 |

**Supplementary Table 2.** Primers sequences for qPCR.

| Gene  (Accession number) | Direction | Sequences (5'-3') (Tm) | Ampli-cation （bp） |
| --- | --- | --- | --- |
| *Actb*  *NM_007393.5* | forward | GGCTGTATTCCCCTCCATCG (59.96℃) | 154 |
|  | reversed | CCAGTTGGTAACAATGCCATGT (59.44℃) |  |
| *Ocn*  *NM_001032298.3* | forward | CTGACCTCACAGATCCCAAGC (60.41℃) | 187 |
|  | reversed | TGGTCTGATAGCTCGTCACAAG (59.84℃) |  |
| *Runx2*  *NM_001271630.2* | forward | GACTGTGGTTACCGTCATGGC (61.27℃) | 84 |
|  | reversed | ACTTGGTTTTTCATAACAGCGGA (59.11℃) |  |
| *Sp7*  *XM_006520519.5* | forward | GGAAAGGAGGCACAAAGAAGC (59.73℃) | 218 |
|  | reversed | CCCCTTAGGCACTAGGAGC (58.86℃) |  |
| *Alpl*  *NM_001287172.2* | forward | CCAACTCTTTTGTGCCAGAGA (58.42℃) | 110 |
|  | reversed | GGCTACATTGGTGTTGAGCTTTT (59.99℃) |  |
| *Col1a1*  *NM_007742.4* | forward | GCTCCTCTTAGGGGCCACT (60.69℃) | 103 |
|  | reversed | CCACGTCTCACCATTGGGG (60.38℃) |  |
| *Scx*  *NM_198885.3* | forward | CTGGCCTCCAGCTACATTTCT (59.79℃) | 237 |
|  | reversed | GTCACGGTCTTTGCTCAACTT (59.06℃) |  |
| *Tnmd*  *NM_022322.2* | forward | ACACTTCTGGCCCGAGGTAT (60.62℃) | 154 |
|  | reversed | GACTTCCAATGTTTCATCAGTGC (58.52℃) |  |
| *Mkx*  *NM_177595.4* | forward | GGGGAGCCGTGCTTTTTGA (60.90℃) | 189 |
|  | reversed | GCCTTACCTTCCCTCCATTCTG (60.42℃) |  |
| *IL1A*  *NM_010554.4* | forward | CGAAGACTACAGTTCTGCCATT (58.41℃) | 126 |
|  | reversed | GACGTTTCAGAGGTTCTCAGAG (58.43℃) |  |
| *IL1B*  *NM_008361.4* | forward | GCAACTGTTCCTGAACTCAACT (59.05℃) | 89 |
|  | reversed | ATCTTTTGGGGTCCGTCAACT (59.58℃) |  |
| *IL6*  *NM_031168.2* | forward | TAGTCCTTCCTACCCCAATTTCC (59.22℃) | 76 |
|  | reversed | TTGGTCCTTAGCCACTCCTTC (59.37℃) |  |
| *Cxcl1*  *NM_008176.3* | forward | CTGGGATTCACCTCAAGAACATC (59.06℃) | 117 |
|  | reversed | CAGGGTCAAGGCAAGCCTC (60.68℃) |  |
| *Cxcl2*  *NM_009140.2* | forward | CCAACCACCAGGCTACAGG (60.00℃) | 108 |
|  | reversed | GCGTCACACTCAAGCTCTG (58.85℃) |  |
| *Cxcl5*  *NM_009141.3* | forward | GTTCCATCTCGCCATTCATGC (60.00℃) | 103 |
|  | reversed | GCGGCTATGACTGAGGAAGG (60.25℃) |  |
| *Cxcl9*  *NM_008599.4* | forward | GGAGTTCGAGGAACCCTAGTG (59.52℃) | 82 |
|  | reversed | GGGATTTGTAGTGGATCGTGC (59.06℃) |  |
| *Cxcl10*  *NM_021274.2* | forward | CCAAGTGCTGCCGTCATTTTC (60.67℃) | 157 |
|  | reversed | GGCTCGCAGGGATGATTTCAA (61.02℃) |  |
| *Cxcl11*  *NM_019494.1* | forward | GGCTTCCTTATGTTCAAACAGGG (59.81℃) | 108 |
|  | reversed | GCCGTTACTCGGGTAAATTACA (58.48℃) |  |
| *Cxcl12*  *NM_001012477.2* | forward | TGCATCAGTGACGGTAAACCA (59.93℃) | 146 |
|  | reversed | TTCTTCAGCCGTGCAACAATC (59.73℃) |  |
| *Cxcl13*  *NM_018866.3* | forward | GGCCACGGTATTCTGGAAGC (61.10℃) | 108 |
|  | reversed | GGGCGTAACTTGAATCCGATCTA (60.24℃) |  |
| *Cxcl14*  *M_019568.2* | forward | GAAGATGGTTATCGTCACCACC (58.80℃) | 116 |
|  | reversed | CGTTCCAGGCATTGTACCACT (60.61℃) |  |
| *Cxcl15*  *NM_011339.2* | forward | CAAGGCTGGTCCATGCTCC (60.75℃) | 183 |
|  | reversed | TGCTATCACTTCCTTTCTGTTGC (59.25℃) |  |
| *Cxcl16*  *NM_023158.7* | forward | CCTTGTCTCTTGCGTTCTTCC (59.20℃) | 139 |
|  | reversed | TCCAAAGTACCCTGCGGTATC (59.52℃) |  |
| *Cxcl17*  *NM_153576.2* | forward | AGGTGGCTCTTGGAAGGTG (59.24℃) | 214 |
|  | reversed | GGTGACATCGTTTGAGAAATTGC (59.09℃) |  |
